# Supplementary material for: Some Preliminary Results to Eradicate Leukemic Cells in Extracorporeal Circulation by Actuating Doxorubicin-Loaded Nanochains of Fe3O4 Nanoparticles
Source: Cells. 2022 Jun 23;11(13):2007. doi: 10.3390/cells11132007 (PMC9265363; doi:10.3390/cells11132007)
Supplement: Supplementary file 1 [file cells-11-02007-s001.zip › cells-1683858-supplementary.pdf]

Supporting Information

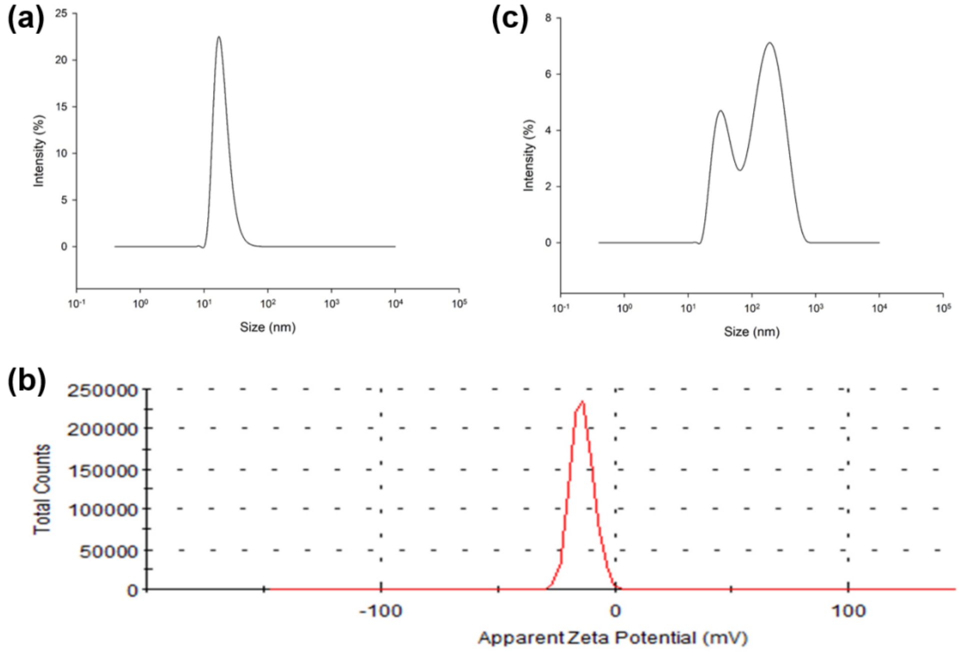

Figure S1. (**a**) Hydrodynamic size of individual oleic acid@Fe_3O_4 nanoparticles. (**b**) Zeta potential of nanochains. (**c**) Hydrodynamic size of nanochains.

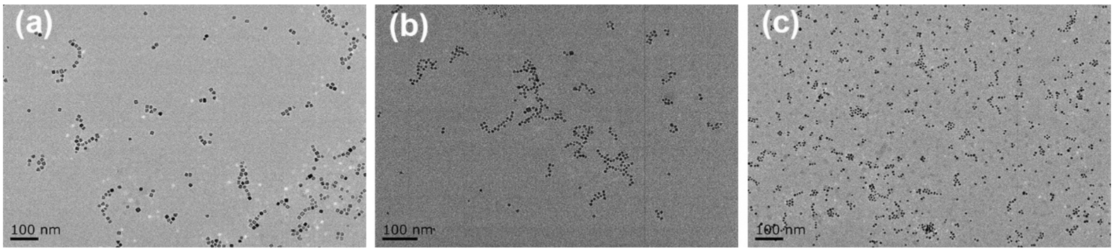

Figure S2. TEM images of nanochains fabricated with different polymers. (**a**) nanochains modified by PLA-PEG. (**b**) nanochains by addition of Tween 80 as stabilizing agents. (**c**) nanochains by addition of F68 on basis of (**b**).

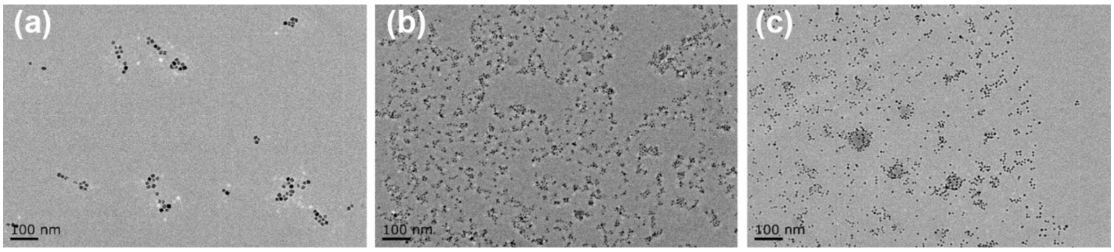

Figure S3. TEM images of nanochains fabricated with different polymer amount. The amounts of OA@Fe_3O_4 nanoparticles were 2mg. (**a**) nanochains fabricated by 50mg PLA-PEG. (**b**) nanochains fabricated by 80mg PLA-PEG. **(c)** nanochains fabricated by 100mg PLA-PEG.

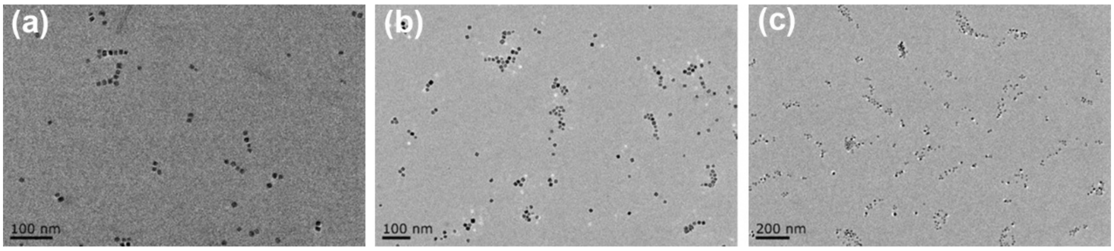

Figure S4. TEM images of nanochains fabricated by different magnetic field intensity and emulsification time. (**a**)–(**c**) The intensity of magnetic field was 40mT, 80mT and 160mT, respectively.

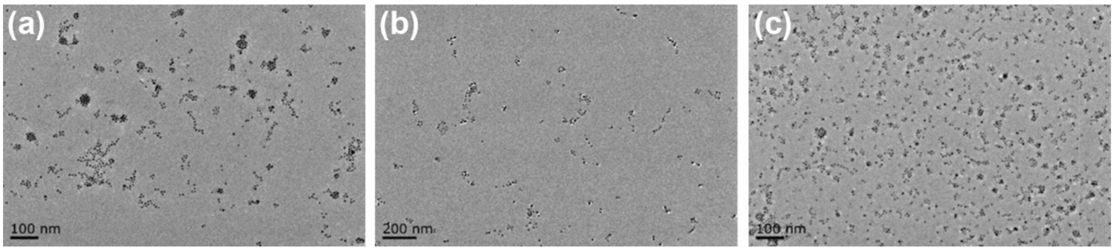

Figure S5. TEM images of nanochains fabricated by different magnetic field

intensity and emulsification time. (**a**)–(**c**) The emulsification time was 3min, 5min and 10min, respectively.

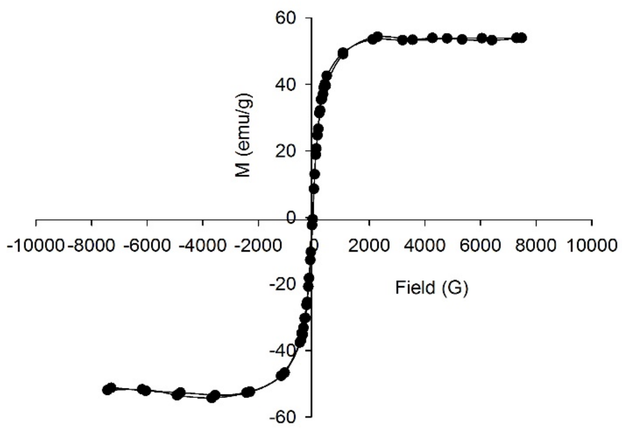

Figure S6. Hysteresis loop of magnetic nanochains.

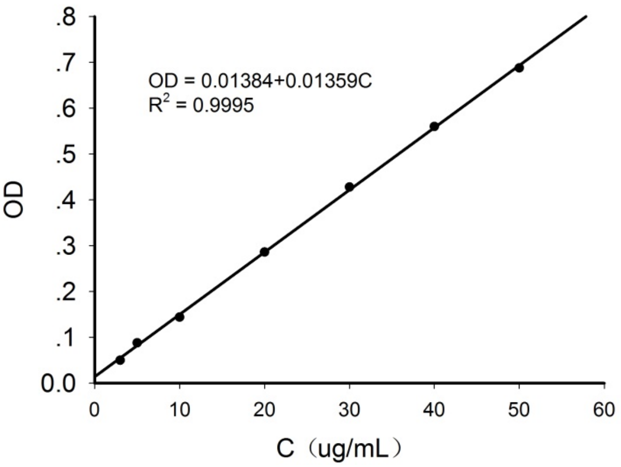

Figure S7. The calibration curve of DOX in determining the release profile of the nanochains.

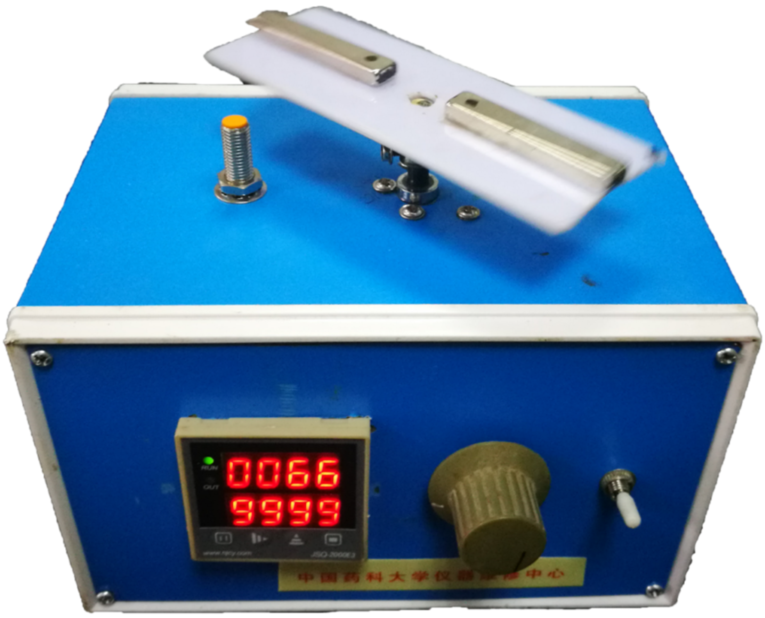

Figure S8. Exterior appearance of the packaged magnetic field generator.

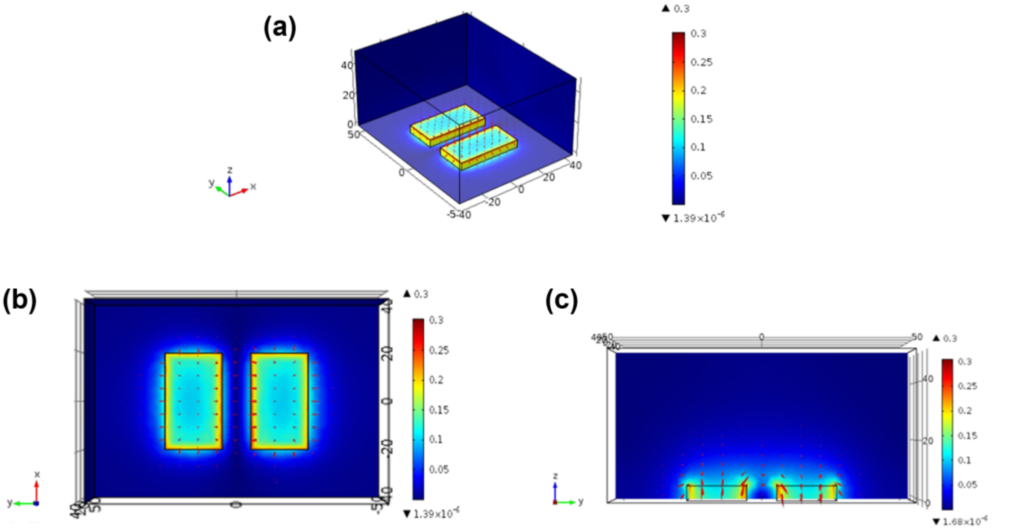

Figure S9. Simulation of the rotational magnetic field. (**a**) Simulation stereogram of the magnetic field. (**b**) Cross-sectional diagram of the magnetic field in xy plane. (**c**) Cross-sectional diagram of the magnetic field in yz plane.

(a)

Table 1 Average hemolysis rate under different conditions and different time periods

Average hemolysis rate of each concentration in different time periods/%	50 μg/mL	100 μg/mL	200 μg/mL
1 h	**3.76**±0.07	**3.78**±0.07	**2.76**±0.05
2 h	**6.98**±0.06	**6.54**±0.06	**2.60**±0.05
3 h	**3.87**±0.05	**3.94**±0.07	**2.00**±0.03

(b)

Table 2 Blood clotting time under different in vitro test conditions

Number	Blood volume	Diluted solution	Final iron concentration	Clotting time
1	20 μL	-	-	2min37s
2	20 μL	20μL normal saline	-	3min16s
3	20 μL	20μL normal saline	200	2min38s
4	20 μL	20μL normal saline	100	2min41s
5	20 μL	20μL normal saline	50	2min47s
6	20 μL	20μL heparin sodium saline	200	>60min
7	20 μL	20μL heparin sodium saline	100	>60min
8	20 μL	20μL heparin sodium saline	50	>60min
9	20 μL	20μL heparin sodium saline	-	>60min

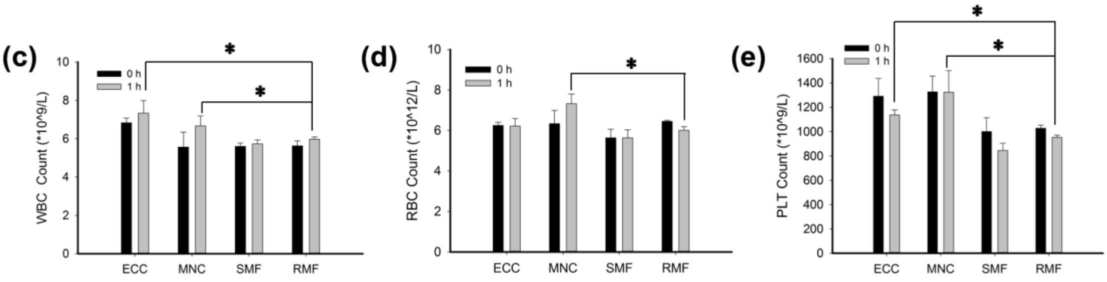

Figure S10. Effects of nanochains upon blood. (**a**) Average hemolysis rate under different condition and different time. (**b**) Blood clotting time *in vitro* under different testing condition. (**c**)–(**e**) Cellular counting of blood cells before and after the extracorporeal circulation. (**c**) White blood cells. (**d**) Red blood cells. (**e**) Blood platelets. *, $p < 0.05$.

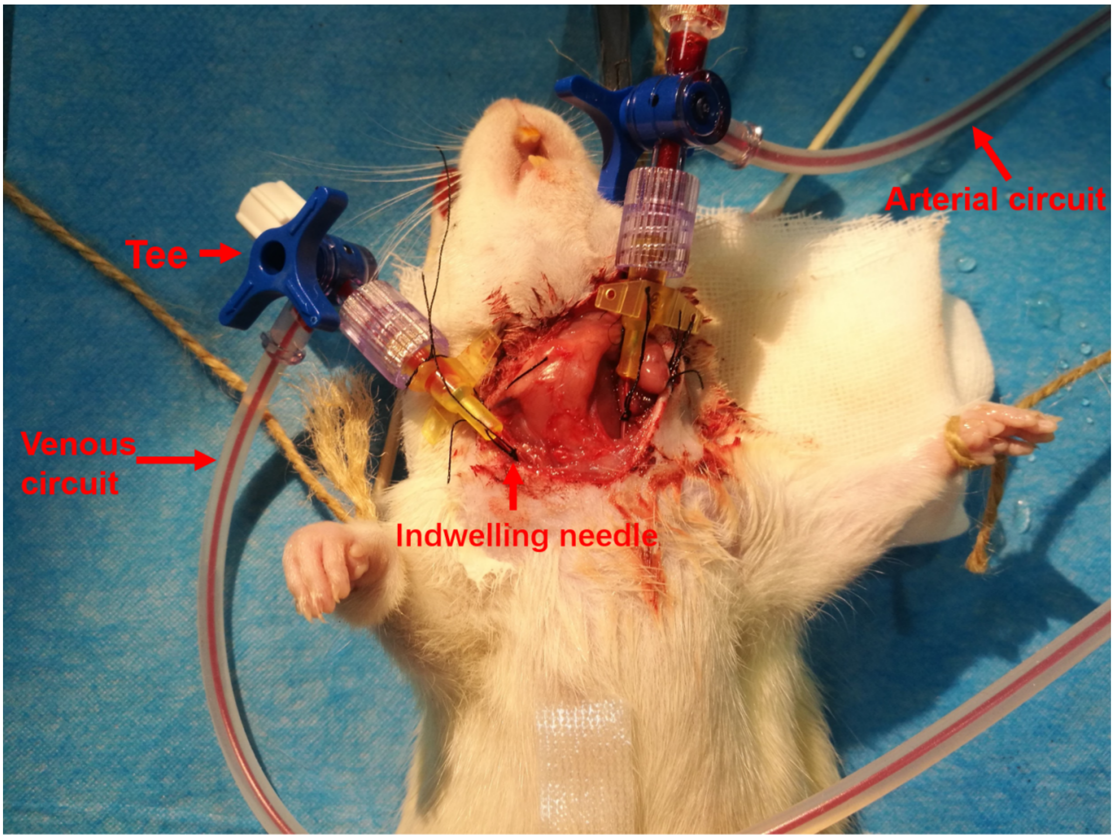

Figure S11. One typical picture of the surgical operation in a rat to form the extracorporeal circulation.
